# Doctors' Cars at the Motor Show

**Published:** 1908-12-12

**Authors:** 


### Motoring Notes.

### DOCTORS' CARS AT THE MOTOR SHOW.

In continuing my description of the cars exhibited at the recent show which are suitable for medical men, I should like to make it clear that I confine myself to the light cars of low or moderate power likely to be of service to the average doctor who cannot afford the luxury and expense of a large multi-cylindered car and a professional chauffeur. Humber, Ltd., of Coventry, whose small Humberettes were so well known a few years ago, are this year putting on the market a new 8-h.p. model. It is a very handy little two-seated car with a twin-cylinder engine and double ignition, accumulator, and high-tension magneto. The water circulation is of the thermo-syphon type, and a multiple disc metal-to-metal clutch is used. The price is £195.

The Anglo-American Motor Car Company exhibited four samples of the well-known 10-h.p. single-cylinder Cadillac, one of which was shown with a special body suitable for a medical man. This is the car that early this year so successfully passed through the very severe "standardisation test" under the auspices of the technical committee of the Royal Automobile Club. The car has two speeds and reverse, with direct drive on the second speed, and, with standard two-seated body, is listed at 195 guineas. The Star Engineering Company, of Wolverhampton, are placing several small cars on the market during the coming season; there are two of the Star class, one 10-h.p. and the other 8-h.p., both with two cylinders, high-tension magneto ignition, pump lubrication, and the latest back axle, priced respectively £210 and £185. This firm is also marketing three small cars of the Starling class, of 6-, 8-, and 10-h.p.; the two former are single-cylinder cars, and the latter a double cylinder. These three models have accumulator ignition and side-chain transmission, and with two-seated body, cost respectively £120, £160, and £175.

The Swift Motor Company, of Coventry, again show their 10-12-h.p. two-cylinder car, which is improved in some details; the gear lever is of the non-binding type, and a floating back axle has taken the place of the former variety, the propeller shaft having universal joints at each end. This car has been deservedly popular with its users, and at the price of £225 with a two-seated body it is decidedly cheap.

The British Motobloc Syndicate, of Shaftesbury Avenue, showed a very smart little doctor's car. The body is two-seated with side doors, and is fitted with a Victoria hood and wind-screen. The petrol and oil tanks are fitted to the dashboard under the bonnet. The engine is of the single-cylinder type (8-9-h.p.), and the combination of engine and gear in one casting has many features to recommend it—the most striking of which is perhaps the efficiency obtained on the road wheels. The ignition is by high-tension magneto, cooling by pump and honeycomb radiator, and the clutch is of the metal-to-metal type. The price of the car complete with hood and screen is £260.

The Darracq Company are this year making a popular model, a two-cylinder 8-10-h.p. car. The ignition is by high-tension magneto, but coil and accumulator can also be fitted at an additional cost of £8. The radiator is of the gilled type, with a gear-driven centrifugal pump. Internal expanding brakes are fitted on the propeller shaft and on the two rear driving-wheels.

Messrs. Renault Frères are another big firm who this year are turning their attention to the small car, and who are showing an 8-h.p. vehicle, which has attracted a considerable amount of attention. It is fitted with a neatly designed two-cylinder engine, the cylinders being cast *en bloc* and thermo-syphon cooling being adopted. In this model, as in all its larger brethren, the radiator is fixed as part of the dashboard, and the bonnet is of the well-known sloping variety. The ignition is by high-tension magneto, and the firm's automatic carburettor is used. The price of the car complete with two-seated body is £200.

The name of Panhard is one to conjure with in motor circles, and Messrs. W. and G. Ducros, of Regent Street, show a new 8-h.p. two-cylinder chassis at the price of £240. This well-known firm has thus come into line with other firms of repute, who have at last realised the wide market there is for a small car at a moderate price. On this model thermo-syphon cooling and magneto ignition are found together with positive lubrication of the engine by means of a pump, of which the two sight feeds are the only dashboard fittings. The clutch is large in size and is of the leather-lined cone type, and the frame is of pressed steel.

Messrs. Adams show their well-known 10-h.p. single-cylinder car with which this company first built up its reputation. It is, however, considerably altered in appearance this year, the bonnet now fitted being longer. The company's new three-speed "always-in-mesh" gear is, of course, fitted. The control by pedals is popular with many people, and as each pedal is definitely marked, a mistake is not easy. At each forward speed the pedal is locked down, but is automatically released when the next is depressed. The ignition is by high-tension magneto or by battery and coil, at the purchaser's option. The price of the car with body complete is £185.

Messrs. G. H. Wait and Co., of Leicester, exhibited a smart 8-10-h.p. two-cylinder car fitted with a White and Poppe engine and Lonquemare carburettor. The transmission is by chain, to which Mr. Wait has been always a faithful adherent. The clutch and change-speed gear are close to the back axle, and the gears are always in mesh. The ignition is by means of accumulator and coil or magneto at option. This little car is one that has gained considerable reputation for hill-climbing, and the price with two-seated body is £200. This firm also build a 6½-h.p. single-cylinder car with two speeds and reverse, the price of this model with two-seated body being £125.

"Viator."

